# Time course study of optical coherence tomography angiography in patients with methanol induced optic neuropathy

**DOI:** 10.1186/s12886-023-02937-x

**Published:** 2023-04-25

**Authors:** Ali Jafarizadeh, Mina Homaie, Mirsaeed Abdollahi, Mohamadreza Niyousha

**Affiliations:** 1 Nikookari Eye Center, Tabriz University of Medical Sciences, Tabriz, Iran; 2 Student Research Committee, Tabriz University of Medical Sciences, Tabriz, Iran; 3 Department of Ophthalmology, Nikookari Eye Center, Tabriz University of Medical Sciences, Tabriz, Iran

**Keywords:** Methanol, Toxic optic neuropathy, Methanol-induced optic neuropathy, Blindness, OCTA, RNFL, Vascular density

## Abstract

**Introduction:**

In countries where alcoholic beverages are legally prohibited, methanol toxicity usually occurs due to ingesting homemade alcoholic drinks. The initial ophthalmologic symptoms of methanol toxicity typically appear 6–48 h after ingestion, and the severity of symptoms varies widely from mild and painless decreased vision to no-light perception vision.

**Methods:**

This prospective study examines 20 patients with acute methanol poisoning within 10 days of use. Patients underwent ocular examinations, BCVA (Best Corrected Visual Acuity) recording, and OCTA (Optical Coherence Tomography Angiography) of the macula and optic disc. BCVA measurement and imaging were repeated one month and three months after intoxication.

**Results:**

There was a statistically significant reduction in superficial parafoveal vascular density (*P*-value = 0.026), inner retinal thickness (*P*-value = 0.022), RNFL (Retinal Nerve Fiber Layer) thickness (*P*-value = 0.031), and an increase in cup to disc ratio (*P*-value < 0.001), and central visual acuity (*P*-value = 0.002) in this time course. However, there was no statistically significant difference in FAZ (Foveal Avascular Zone) area (*P*-value = 0.309), FAZ perimeter (*P*-value = 0.504), FD-300 (Foveal density, vascular density within a 300 μm wide region of the FAZ) (*P*-value = 0.541), superficial vascular density (*P*-value = 0.187), deep foveal vascular density (*P*-value = 0.889), deep parafoveal vascular density (*P*-value = 0.830), choroidal flow area (*P*-value = 0.464), total retinal thickness (*P*-value = 0.597), outer retinal thickness (*P*-value = 0.067), optic disc whole image vascular density (*P*-value = 0.146), vascular density inside the disc (*P*-value = 0.864), or peripapillary vascular density (*P*-value = 0.680) at different times.

**Conclusion:**

Over time, methanol poisoning can cause changes in retinal layers thickness, vasculature, and optic nerve head. The most important changes include cupping of the optic nerve head, reduction in RNFL thickness, and inner retinal thickness.

## Introduction

Methanol toxicity is a significant public health concern in some developing countries. Methanol is a clear, colorless, and flammable liquid metabolized to formaldehyde by alcohol dehydrogenase. Then, formaldehyde is rapidly converted to formic acid, which causes the majority of the toxicity associated with methanol. Methanol is a solvent in printing and reproduction solutions, adhesives, paints, polishes, and stabilizers, and these products are readily available. It is also used as an antifreeze in car windshield washer solutions and as a diesel additive [2]. During the COVID-19 pandemic, methanol poisoning rates increased in some countries, along with the sale of alcohol for disinfection increasing by 14–30% [2]. This relationship seems to be due to increased ease of access to non-standard alcohol disinfectants, raised probability of accidental or intentional consumption of fake alcohol, and some cultural misconceptions [2, 4].

Methanol can cause severe poisoning if taken orally. The amount of 30 ml of methanol can be fatal. Although the lethal dose of methanol is typically between 100–125 ml [5]. In a study by Aghababaeian, of 768 patients poisoned by non-standard alcoholic beverages, 96 (12.5%) patients died from all types of alcoholic poisoning, and 76 patients died specifically (10.1%) due to methanol poisoning [6].

The effects of methanol poisoning are usually limited to the nervous system, vision, and gastrointestinal tract. Visual impairment is the most critical primary symptom of methanol poisoning called MION (Methanol-Induced Optic Neuropathy) and starts 12–24 h after exposure. Visual symptoms can vary from blurred vision to some permanent complications, such as complete blindness in 25–33% of patients [7, 8]. Blurred vision, decreased visual acuity, central scotoma, photophobia, and “snowstorm” are the most common complaints of patients [9]. Ingestion of 10 ml methanol can cause permanent and complete vision loss after 10–30 h of consumption or longer in case of simultaneous consumption with ethanol. This happens due to retrolaminar axonal demyelination, a progressive condition that causes atrophy of the optic nerve [5, 7]. Methanol poisoning in the eye causes optic disc hyperemia, which results from methanol-induced vasodilatation in the retina and brain [10, 11]. Peripapillary retinal and optic disc edema with loss of physiologic cupping usually occur more slowly than disc hyperemia [12]. Within a day, the white lines of edema spread to the retina. The hyperemia of the optic disc usually subsides within three days; however, the retinal edema may remain for several weeks. Diminishing of pathologic changes of the fundus and improvement of visual acuity occurs 1–2 months after poisoning. However, 25–40% of the patients may present long-term visual impairment [13].

Optical Coherence Tomography (OCT) is a non-invasive, high-resolution optical imaging technology that uses interference between a signal from an object under investigation and a local reference signal to produce images. Optical Coherence Tomography Angiography (OCTA) can produce images of blood flow with the resolution of all the retinal vascular layers, diagnosing diseases such as glaucoma, macular degeneration, and diabetic retinopathy [9, 14].

Due to the lack of a similar study, this study aimed to evaluate the course of OCTA changes in 20 patients with methanol toxicity at intervals of 10 days, one month, and three months.

## Materials and methods

This study investigated 20 patients between 15 to 53 years of age with acute methanol poisoning within 10 days of consumption. These patients were referred to Nikookari Ophthalmology Center in Tabriz after receiving the necessary treatments, such as sodium bicarbonate (1–2 meq/kg bolus and 132 meq infusion for patients with PH < 7.3), ethanol (10 mg/kg), folic acid (50 mg every six hours, continued for one month), methylprednisolone (1000 mg daily for three days), erythropoietin (20,000 unit daily for three days), and hemodialysis. Both eyes of each patient were examined, and the eye with better OCTA quality was included in the study. Of 20 eyes, 13 were right eyes, and seven were left. This study started in April 2020 and continued for six months. All patients underwent clinical ophthalmic examinations, including BCVA based on logMAR (Minimum Angle of Resolution), slit lamp examination, detailed fundus examination, and OCTA of the macula and optic nerve head. OCTA was repeated one month and three months after poisoning. Imaging was done by an Opto-Vue OCT device, which can produce images (3 mm × 3 mm) of the macula with a foveal center. Each OCT angiography device contains 304 × 304 pixels resulting from the intersection of vertical and horizontal B-scan images. OCTA images were evaluated by a retina specialist in terms of segmentation accuracy and image quality. OCTA measures the FAZ (Foveal Avascular Zone) area, FAZ perimeter, FD-300 (foveal density, vascular density within a 300 μm wide region of the FAZ), superficial and deep foveal vascular density, superficial and deep parafoveal vascular density, choroidal flow area, total retinal thickness, inner and outer retinal thickness, optic disc whole image vascular density, vascular density inside the disc, peripapillary vascular density, cup to disc ratio, RNFL (Retinal Nerve Fiber Layer) thickness. During the follow-up, The visual acuity of each patient was recorded, and its time course was evaluated.

Exclusion criteria were as follows: patients with a poor general condition, retinal pathologies such as retinal dystrophies, trauma, pathological myopia (spherical equivalence > 6D), history of uveitis, hypertension, diabetic retinopathy and papillopathy, history of retinal surgeries, and patients whose OCTA image did not have the appropriate quality.

Due to the quantitative variables during the time period (three points) and the comparison of these periods, we analyzed the standard deviation and mean using repeated measures one-way ANOVA test. We analyzed the data using IBM SPSS Statistics for Windows, version 23 (IBM Corp., Armonk, N.Y., USA) statistical software and considered the significance level at *P* = 0.05.

In this study, we considered all the ethical principles by confirmation of the ethical committee of Tabriz University of medical science (IR.TBZMED.REC.1399.404). The study was performed in accordance with the Helsinki Declaration. All patients obtained full knowledge of this study by reading the informed consent form and confirming it. Patients were not charged for obtaining OCTA images.

## Results

Twenty patients with methanol poisoning were examined, of which three (15%) were women and 17 (85%) were men. The average age of the patients was 34.96 ± 12.34, the youngest was 15 years and the oldest was 53 years.

According to Table 1 and statistical data and tests, there was no statistically significant difference in FAZ area (*P*-value = 0.309), FAZ perimeter (*P*-value = 0.504), FD-300 (foveal density, vascular density within a 300 μm wide region of the FAZ) (*P*-value = 0.541), superficial vascular density (*P*-value = 0.187), deep foveal vascular density (*P*-value = 0.889), deep parafoveal vascular density (*P*-value = 0.830), choroidal flow area (*P*-value = 0.464), total retinal thickness (*P*-value = 0.597), outer retinal thickness (*P*-value = 0.067), optic disc whole image vascular density (*P*-value = 0.146), vascular density inside the disc (*P*-value = 0.864), peripapillary vascular density (*P*-value = 0.680) at different times. However, there was statistically significant decrease in superficial parafoveal vascular density (*P*-value = 0.026), inner retinal thickness (*P*-value = 0.022), RNFL thickness (*P*-value = 0.031) and central visual acuity (*P*-value = 0.002) and significant increase in cup to disc ratio (*P*-value < 0.001), in time course. In other words, according to our findings and a statistically significant difference in inner retinal thickness three months after poisoning, we have seen a decrease in the retinal thickness between the internal limiting membrane and the external limiting membrane of the patients' eyes. The rise in optic disc cupping parallels the decrease of the retinal nerve fiber layer thickness. Central visual acuity of the patients based on the logMAR decreased, which means relative recovery after poisoning.

**Table 1:** OCTA results and central visual acuity at different times and their related data

Variable	Time	Mean	Standard deviation	*P*-value
**Superficial foveal vascular density (%)**	First 10 days	20.27	$\pm$ 7.65	0.187
	1 month later	21.34	$\pm$ 9.04	
	3 months later	16.22	$\pm$ 10.39	
**Deep foveal vascular density (%)**	First 10 days	40.06	$\pm$ 7.33	0.889
	1 month later	40.88	$\pm$ 8.70	
	3 months later	36.85	$\pm$ 8.06	
**Superficial parafoveal vascular density (%)**	First 10 days	49.03	$\pm$ 3.71	0.026
	1 month later	48.96	$\pm$ 3.81	
	3 months later	45.59	$\pm$ 4.67	
**Deep parafoveal vascular density (%)**	First 10 days	51.75	$\pm$ 3.91	0.830
	1 month later	54.44	$\pm$ 3.64	
	3 months later	56.49	$\pm$ 2.02	
**Optic disc whole image vascular density (%)**	First 10 days	41.93	$\pm$ 3.41	0.146
	1 month later	41.38	$\pm$ 4.01	
	3 months later	38.67	$\pm$ 7.58	
**Vascular density inside the disc (%)**	First 10 days	43.20	$\pm$ 3.05	0.864
	1 month later	42.64	$\pm$ 4.25	
	3 months later	43.12	$\pm$ 6.00	
**Peripapillary vascular density (%)**	First 10 days	41.98	$\pm$ 4.21	0.680
	1 month later	41.66	$\pm$ 4.83	
	3 months later	39.89	$\pm$ 9.16	
**Foveal avascular zone area (mm**^**2**^**)**	First 10 days	0.20	$\pm$ 0.06	0.309
	1 month later	0.19	$\pm$ 0.07	
	3 months later	0.21	$\pm$ 0.08	
**Foveal avascular zone perimeter**	First 10 days	1.78	$\pm$ 0.27	0.540
	1 month later	1.75	$\pm$ 0.36	
	3 months later	1.78	$\pm$ 0.38	
**Foveal density (FD-300)**	First 10 days	49.69	$\pm$ 3.92	0.541
	1 month later	51.99	$\pm$ 3.09	
	3 months later	50.49	$\pm$ 3.39	
**Choroidal flow area (mm**^**2**^**)**	First 10 days	0.49	$\pm$ 0.30	0.464
	1 month later	0.46	$\pm$ 0.25	
	3 months later	0.42	$\pm$ 0.21	
**Total retinal thickness (µm)**	First 10 days	253.64	$\pm$ 14.42	0.597
	1 month later	253.36	$\pm$ 18.68	
	3 months later	246.00	$\pm$ 19.91	
**Inner retinal thickness (µm)**	First 10 days	46.82	$\pm$ 13.13	0.022
	1 month later	47.91	$\pm$ 14.10	
	3 months later	35.91	$\pm$ 19.34	
**Outer retinal thickness (µm)**	First 10 days	206.82	$\pm$ 11.41	0.067
	1 month later	205.18	$\pm$ 11.49	
	3 months later	210.09	$\pm$ 12.62	
**Cup to disc ratio**	First 10 days	0.15	$\pm$ 0.11	0.001 $>$
	1 month later	0.14	$\pm$ 0.12	
	3 months later	0.32	$\pm$ 0.22	
**Retinal nerve fiber layer thickness (µm)**	First 10 days	116.00	$\pm$ 36.69	0.031
	1 month later	113.91	$\pm$ 41.05	
	3 months later	74.73	$\pm$ 42.71	
**Central visual acuity (logMAR)**	First 10 days	2.70	$\pm$ 1.91	0.002
	1 month later	1.11	$\pm$ 0.56	
	3 months later	1.02	$\pm$ 0.69	

## Discussion

Methanol poisoning can be lethal and has catastrophic visual effects. The recent methanol poisoning outbreak during the Covid-19 pandemic was the most significant methanol poisoning in Iran and the world. Visual symptoms due to methanol poisoning might appear hours after ingestion, ranging from a progressive decrease in vision to dyschromatopsia, scotoma, and photophobia [15].

Similar to previous studies, 80% of poisoned patients were males, while 20% were females [6, 16]. The higher occurrence of poisoning in men may be due to the more frequent consumption of alcoholic beverages in greater volumes than in women [17]. Another reason to be considered is more risky behaviors in men [18].

**Fig. 1:**
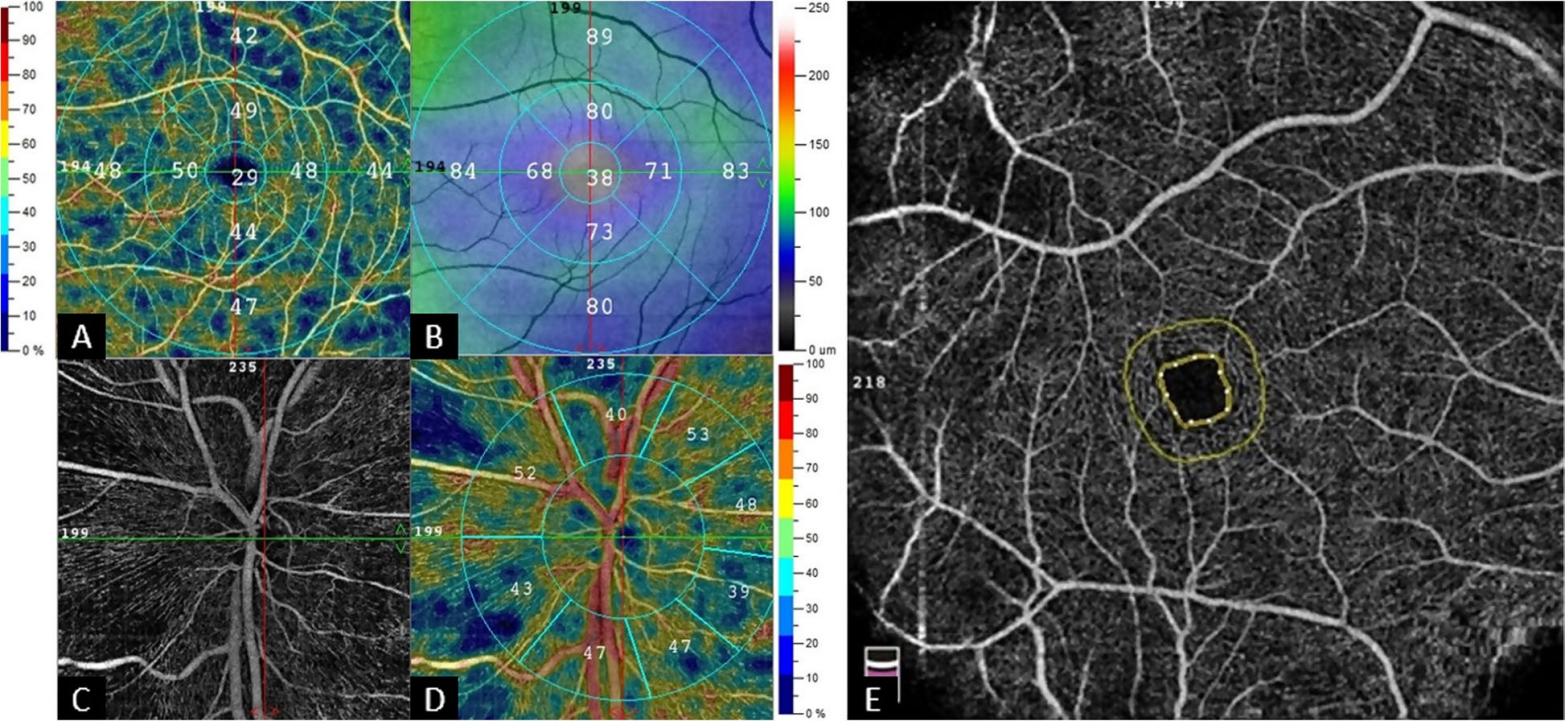
OCTA images (**A** Superficial vessel dinsity, **B** Inner thickness, **C** radial peripapillary capillary, **D** radial peripapillary capillary vessel density, **E** foveal avascular zone)

We found that superficial parafoveal vascular density decreased in patients with methanol toxicity, but there were no significant changes in deep parafoveal, peripapillary, and inside disc vascular density. On the other hand, there was a significant decrease in inner retinal thickness, which can be a reason for the lower superficial parafoveal vascular density. However, Abri Aghdam et al. [19] reported that inside disc and peripapillary vessel density were significantly lower in patients with methanol-induced optic neuropathy compared to normal individuals after six months of poisoning. These differences may be due to population size and length of follow-up, and age. Nevertheless, the cause of this distribution of changes in different regions of the retina is unclear and more studies are recommended in this field (Fig. 1).

In two studies by Klein and Hedges [2] and Shin and Uhm [20] the RNFL thickness after methanol poisoning decreased bilaterally around the optic disc, and as the axonal inflammation resolved, the RNFL got thin. Nurieva et al. [21] showed that ethanol administration and a higher rate of formate elimination were associated with higher RNFL thickness. The present study confirmed previous reports that the RNFL thickness significantly decreases after poisoning. In a recent study, the cause of this decrease was attributed to changes in the optic disc and radial peripapillary capillary (RPC) vessel density [19]. According to Galvez-Ruiz et al. [16], all patients with methanol toxicity had optic nerve atrophy, and almost half of the patients had cupping (cup to disc ratio) more than 0.8 at least in one eye after one month. Cup to disc ratios in our study increased significantly in three months. However, there was no increase in the first month after poisoning, and the means of the cup to disc ratio of the patients' eyes in three months follow-up was lower than 0.8, but it may increase with longer follow-ups. One of the possible causes of cupping in severe methanol poisoning can be due to the toxic effect of methanol metabolites such as formic acid on the mitochondria of prelaminar astrocytic glial cells and retrolaminar oligodendrocytes, which results in the loss of axons and other cells in the prelaminar region [16]. Loss of axons and cells in the prelaminar region in arteritic AION disease also leads to cupping. However, the loss of axons and cells in arteritic AION disease is due to ischemia, but in methanol poisoning, it is caused by mitochondrial dysfunction due to toxic metabolites [22]. In mild cases, cupping occurs less often, which is due to the insufficient amount of toxic substances to destroy the substantial astrocyte cells [23]. The central visual acuity examination in the three-month follow-up showed improvement relative to the acute phase, which may be due to the detxification. Furthermore, the central vision relative to the peripheral was improving because the central ganglion cells are more resistant than peripheral cells. With long-term follow-up, the final central visual acuity will probably get worse and decrease. Nurieva et al. [21] reported that acute retinal ganglion cell injury by methanol was followed by chronic neurodegeneration and progressive axonal loss in up to 25% of the patients.

Limitations of this study were its small sample size, lack of analysis between different retinal and demographic variables, short study duration, lack of investigation and analysis of the severity of poisoning and the blood level of methanol and its metabolites with clinical results and OCTA changes. Also, in this study, it was not possible to consider confounders. Therefore, studies with larger sample sizes and a more extended follow-up period are required to obtain better results with greater reliability.

## Conclusions

Methanol poisoning can cause changes in the retinal layers and vessels and the optic nerve head. Optic nerve head cupping, reduction in RNFL thickness and inner retinal thickness are the most important results of this study. These changes happened over time, so it is necessary to follow up the patients for longer.

## Data Availability

We present all available data in this article and if more explanations are needed, contact the corresponding author.

## Abbreviations

BCVA: Best corrected visual acuity
OCTA: Optical coherence tomography angiography
RNFL: Retinal nerve fiber layer
FAZ: Foveal avascular zone area
FD-300: Foveal density, vascular density within a 300 μm wide region of the FAZ
COVID-19: Coronavirus Disease 2019
MION: Methanol-induced optic neuropathy
OCT: Optical coherence tomography
OCTA: Optical coherence tomography angiography
RPC: Radial peripapillary capillary
